# Peri-device leak and late massive thrombus formation after left atrial appendage occlusion: A case report

**DOI:** 10.1097/MD.0000000000046199

**Published:** 2025-12-26

**Authors:** Yi-Ting Chen, Yung-Lung Chen, Chiung-Jen Wu, Hui-Ting Wang

**Affiliations:** a Division of Cardiology, Department of Internal Medicine, Kaohsiung Chang Gung Memorial Hospital, Chang Gung University College of Medicine, Kaohsiung, Taiwan; b Department of Emergency Medicine, Kaohsiung Chang Gung Memorial Hospital, Chang Gung University College of Medicine, Kaohsiung, Taiwan; c School of Medicine, College of Medicine, National Sun Yat-sen University, Kaohsiung, Taiwan.

**Keywords:** atrial fibrillation, device-related thrombus, left atrial appendage occlusion, oral nticoagulation, peri-device leak

## Abstract

**Rationale:**

Stroke prevention in patients with non-valvular atrial fibrillation who cannot tolerate long-term anticoagulation remains a major clinical challenge. Left atrial appendage occlusion (LAAO) offers an alternative strategy to reduce thromboembolic risk, but device-related thrombus (DRT) formation can undermine its protective benefit. Although most DRT occur early, late thrombus formation related to minimal peri-device leak (PDL) is increasingly recognized. Understanding this rare but serious complication is crucial for optimizing long-term post-LAAO management.

**Patient concerns:**

A 65-year-old male with prior ischemic stroke presented to the emergency department 10 months after LAAO with acute dizziness but no neurological deficits.

**Diagnoses:**

Transesophageal echocardiography and cardiac computed tomography angiography revealed a large thrombus (3.8 × 2.4 cm) on the Watchman device with minimal PDL, consistent with massive late DRT.

**Interventions:**

The patient was started on high-intensity warfarin therapy (international normalized ratio: 2.5–3.5) for anticoagulation. Clopidogrel was temporarily discontinued to reduce bleeding risk.

**Outcomes:**

After 2 months of warfarin therapy, repeat cardiac imaging confirmed complete thrombus resolution. The patient remained neurologically intact without embolic complications.

**Lessons:**

Even minimal PDL can serve as a nidus for significant late thrombus formation, particularly in patients with interrupted anticoagulation. This case highlights the importance of continued long-term imaging surveillance after LAAO and individualized anticoagulation strategies. Clinicians should maintain vigilance for late DRT, especially in high-risk patients, and consider extended follow-up protocols to optimize outcomes.

## 1. Introduction

Left atrial appendage occlusion (LAAO) is an established strategy for preventing thromboembolic stroke in patients with nonvalvular atrial fibrillation (AF) who are contraindicated for long-term oral anticoagulation. Device-related thrombus (DRT) is a notable, serious complication of LAAO with a reported incidence of 1.5% to 14.0% because of the variability in the frequency and standardization of post-LAAO surveillance imaging.^[1]^ It is associated with a significantly elevated risk of ischemic stroke and systemic embolism.^[2]^ The risk factors of DRT include patient factors, procedural factors, and post-procedural antithrombotic management.^[3]^ Prior stroke or transient ischemic attack and permanent AF were the most consistent patient-related DRT predictors, while implantation depth was the most consistent procedural risk factor.^[4,5]^ In addition, low velocities (<0.2 m/s), eddies or stagnated flow patterns, and high endothelial cell activation potential (indicative of complex blood flow patterns, low wall shear stress, and areas more prone to thrombi) are risk factors for DRT after LAAO.^[6]^ Peri-device leak (PDL) typically refers to any active, unprotected communication between the LA and the LAA body beyond the LAAO device, as exhibited on transesophageal echocardiography (TEE) or cardiac computed tomography angiography (CTA).^[7]^ Although any device leak < 5 mm was considered benign and nonsignificant in the landmark randomized controlled trials,^[8]^ pooled data of 1054 patients from the PROTECT AF, PREVAIL (Evaluation of the Watchman LAA Closure Device in Patients with AF vs Long Term Warfarin Therapy), and CAP (Continued Access to PROTECT AF) studies showed that 27.7% of patients had PDL < 5 mm at 1 year, which was associated with an increased 5-year risk for ischemic stroke and systemic embolism (adjusted HR: 1.94; *P* = .014).^[9]^ Image follow-ups 45 days, 3 months and 12 months were suggested after LAAO to detect early DRT formation, and repeated 45 days to 3 months follow-up were suggested in patients with PDL and DRT detected during the previous image study to guide treatment decision.^[3,10,11]^ We present the case of a 65-year-old male with a minimal PDL (<3 mm) after LAAO who developed massive late DRT on a Watchman LAA occluder 10 months after implantation.

## 2. Case report

**Figure 1.**
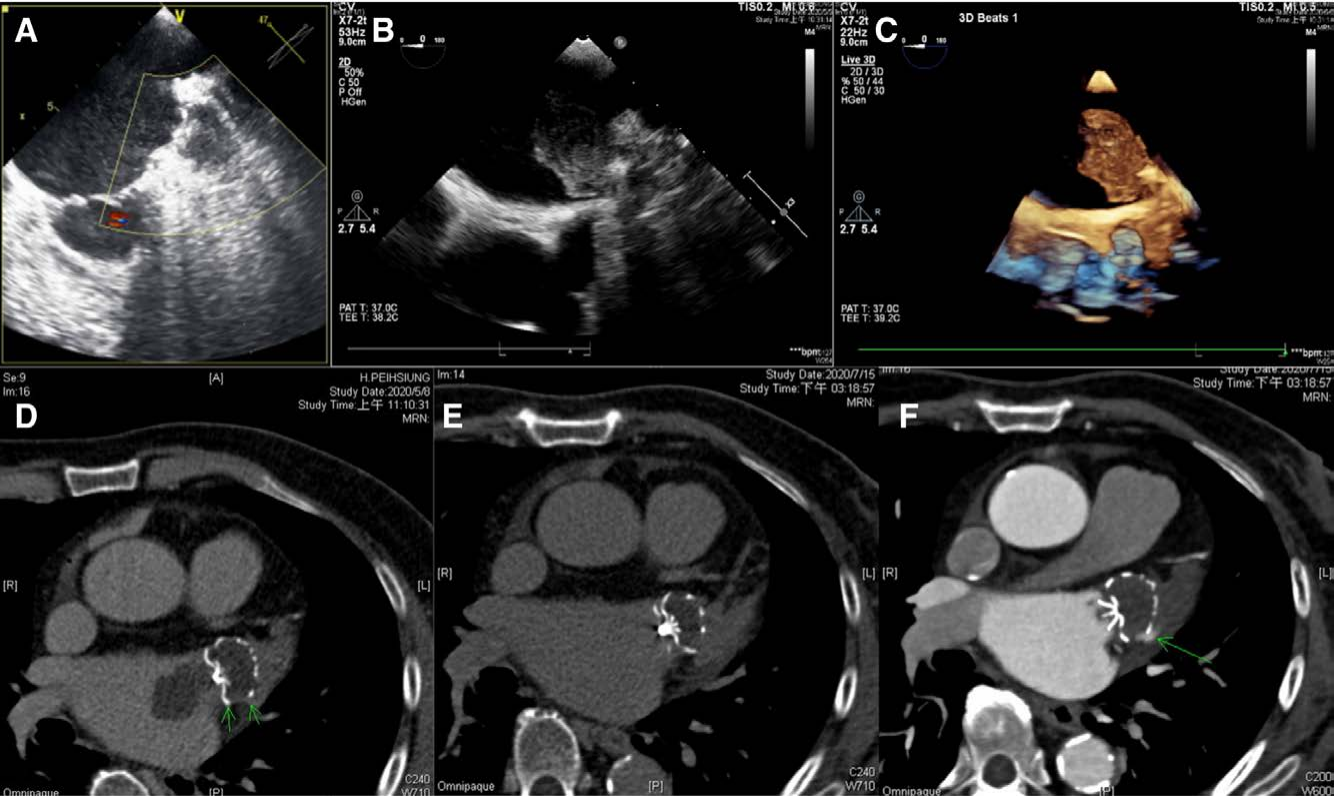
Imaging of left atrial appendage occlusion follow-up. A: TEE 45 days after left atrial appendage occlusion demonstrating a clear Watchman device surface without thrombus; B and C: Two-dimensional and three-dimensional TEE 10 months later showed a large immobile thrombus (2.3 × 4.5 cm) attached to the device surface, remarkable spontaneous echo contrast, and dilated left atrium; D: Cardiac CT angiography confirmed thrombus (3.8 × 2.4 cm) and a minimal peri-device leak; E and F Non-contrast and contrast-enhanced cardiac CT angiography after 2 months of anticoagulation therapy demonstrated complete thrombus resolution and stable minimal leakage. CT = computed tomography, TEE = transesophageal echocardiography.

A 65-year-old male presented to the emergency department with acute onset of dizziness. Ten months prior to presentation, the patient had undergone percutaneous LAAO with a 33-mm Watchman device because of a high risk of stroke from non-valvular AF. After device implantation, the patient received dual antithrombotic therapy with rivaroxaban (15 mg once daily) and clopidogrel. Routine TEE 45 days after implantation confirmed that the occluder was well-positioned with no thrombi (Fig. 1A). However, rivaroxaban was discontinued 5 days later by the treating physician after TEE image because of bleeding complications (frequent genitourinary bleeding manifested as hematuria during rivaroxaban monotherapy before LAAO, and there were still 2 bleeding episodes after LAAO under rivaroxaban and clopidogrel dual therapy). No specific bleeder or malignancy was found by repeated cystoscopy. The patient continued clopidogrel monotherapy. He remained clinically stable until the current presentation, when he developed acute dizziness without syncope or focal neurological symptoms. The patient’s medical history was notable for recurrent ischemic stroke due to AF, which was an indication for LAAO. He also had a history of bleeding during anticoagulation treatment (including hematuria while on rivaroxaban for stroke prevention). No other significant comorbidities were noted. He had no notable personal habits such as tobacco or excessive alcohol use. There was no known family history of AF, stroke, or bleeding disorders. Upon examination at presentation, the patient was alert with stable vital signs. Cardiovascular and respiratory examinations were unremarkable. A neurological examination revealed no focal deficits. Overall, the physical examination results were within normal limits, except for the complaint of dizziness. Initial laboratory investigations, including complete blood count, renal function, electrolyte levels, and coagulation profile, were within normal limits. There was no evidence of infection or other acute metabolic disturbances that could explain the dizziness symptoms.

Given his history of LAAO, an urgent TEE was performed upon presentation. The TEE revealed a large, immobile thrombus measuring 2.3 × 4.5 cm attached to the atrial side of the Watchman device. There was also a dense spontaneous echo contrast in the markedly dilated left atrium (Fig. 1B and C). Cardiac CTA was performed for further evaluation and confirmed a thrombus (3.8 × 2.4 cm) on the device surface. Minimal peridevice leak was also identified (Fig. 1D). The Watchman device remained correctly positioned, with no evidence of dislodgement. The final diagnosis was massive late DRT on the LAA occluder (Watchman 33 mm device) with minimal peridevice leakage. Aggressive anticoagulation therapy was initiated to treat the DRT. Heparin was administered intravenously and then switched to oral warfarin. Warfarin was titrated to a high-intensity therapeutic range [target international normalized ratio (INR): 2.5–3.5]. Prior antiplatelet therapy (clopidogrel) was temporarily discontinued to reduce the bleeding risk while on full anticoagulation.

After the treatment, the patient’s dizziness resolved, and no new neurological events occurred. Both non-contrast and contrast-enhanced cardiac CTA scans at 2 months showed no residual thrombus and a stable, trivial peridevice leak (Fig. 1E and F). Owing to the successful resolution of the thrombus, the patient continued anticoagulation for at least 3 months in total with close monitoring and received ongoing periodic surveillance imaging because of the elevated risk of thrombus recurrence. At the 3-month follow-up, repeat TEE confirmed sustained thrombus resolution on the device surface and persistent minimal PDL (<3 mm). Clinically, the patient remained asymptomatic, with no recurrent neurological or embolic events and no bleeding complications throughout a 6-month observation period. Laboratory testing showed stable coagulation parameters, and no adverse events related to high-intensity warfarin therapy were recorded.

## 3. Discussion

This case illustrates a rare but important complication of LAAO: the formation of a large, late-occurring thrombus on the occlusion device in the presence of a minimal PDL. DRT is not an uncommon complication following LAAO. The newest meta-analysis data, which included 59 eligible studies, showed that the incidence of DRT was 366/12,845 (2.8%, ranging from 0–11%, *I*^2^ = 64%).^[12]^ Patients with DRT are at a greater risk of ischemic stroke or transient ischemic attack than those without DRT.^[13]^ Accordingly, prompt detection and treatment of DRT are critical to prevent thromboembolic events.

In patients with an LAA occluder, thrombi detection relies on imaging modalities, primarily TEE and cardiac CTA. TEE is the standard for routine surveillance owing to its excellent visualization of the device surface and high specificity for thrombi. Although TEE remains the standard, cardiac CTA offers superior spatial resolution and reliable detection of DRT. It has comparable efficacy to TEE with a higher sensitivity for detecting subtle thrombus formation.^[14]^ In this case, TEE identified a sizable thrombus, and cardiac CTA was used to confirm the findings and characterize the extent of the thrombus and PDL. Cardiac CTA has a complementary role in confirming DRT and evaluating associated findings such as residual leak.

An intriguing aspect of our patient’s presentation was that only a trivial peridevice leak was present, yet a massive thrombus still formed on the device. PDL refers to the residual blood flow around an implanted appendage occluder. In the original Watchman device trials, a residual jet of ≤ 5 mm on color Doppler was considered an acceptable result for successful LAA closure.^[9]^ Early post hoc analyses from the PROTECT-AF trial suggested that small leaks (≤5 mm) were common and were not associated with a higher thromboembolic risk. The PROTECT-AF sub-study demonstrated that patients with minor leaks did not experience more strokes than those with complete closure, although the low event rates indicated that the analysis was underpowered. As a result, a PDL of ≤ 5 mm was deemed clinically insignificant.^[15]^

However, recent evidence challenges this conclusion. Growing data indicate that even minimal leaks are associated with an elevated risk of thromboembolism. A pooled 5-year analysis of Watchman 2.5 device trials and registries found that 28% of patients had a ≤ 5 mm leak at 1 year. The presence of these small leaks was associated with an almost doubled rate of ischemic stroke or systemic embolism compared to no leak (adjusted hazard ratio: 1.94).^[14]^ Similarly, a large analysis of 51,333 LAAO cases from the National Cardiovascular Data Registry showed that small (0–5 mm) leaks at 45 days were linked to modest but significantly higher odds of stroke/transient ischemic attack or systemic embolization (15% relative increase), as well as a slightly increased bleeding risk.^[16,17]^ Patients with larger leaks (>5 mm) did not show higher event rates than those without. This finding is attributed to more aggressive anticoagulation treatment in those with large leaks (44.9% remained on warfarin vs 33.0% of those with no or small leaks).^[16]^ In addition, studies have demonstrated that 8% to 14% of patients with PDL < 3 mm can progress to severe leaks at follow-up, thus highlighting that frequent device surveillance is vital.^[18]^

Therefore, even minor leaks may not be entirely benign. Any residual leaks should be monitored closely. Persistent small leaks beyond the initial healing period warrant consideration for prolonged anticoagulation treatment or percutaneous PDL closure if significant, as clinically appropriate in certain cases. Our case reinforced that a minimal residual leak can serve as a nidus for late thrombus formation on an occlusion device, particularly if anticoagulation therapy is discontinued.

Other device- and patient-related factors can contribute to DRT risk following LAAO. The first-generation Watchman 2.5 device, which was used in our patient, has an exposed metal screw hub on its atrial face that can act as a nidus for thrombus formation if not fully endothelialized.^[4]^ Many device-related clots originated in the central hub region. Incomplete or delayed endothelialization of any portion of the device (e.g., due to impaired healing or ongoing inflammation) can facilitate thrombus formation on the device surface. Deep implantation of the device is another recognized risk factor. If the occluder is deployed too distal to the inside of the appendage, the ostium may not be fully sealed and leaves a rim of exposed atrial appendage tissue that remains in contact with circulating blood.^[4,19]^ This situation promotes clot formation at the interface between the device and native tissue. A device that is overly large relative to the LAA anatomy can also increase the risk owing to the greater surface area of the exposed device.

Patient-specific anatomical and physiological factors (e.g., a very large or multilobed LAA cavity or low left atrial flow velocities in heart failure with reduced ejection fraction) are associated with stasis and thrombus formation on LAAO devices.^[19,20]^ In our patient, the presence of a markedly dilated left atrium (indicating low flow and stasis) combined with the interruption of anticoagulation likely created a prothrombotic milieu conducive to late thrombus development on the device surface.

Because DRT is often asymptomatic, emphasis must be placed on surveillance after LAAO. Expert consensus guidelines generally advise performing TEE 45 days after implantation (our patient followed this recommendation) and then again around 3 months (6–12 weeks) after the procedure to check for thrombus formation and device issues.^[7]^ Early identification of DRT allows the initiation of prompt anticoagulation therapy before the occurrence of a stroke or systemic embolus. In the present case, no thrombus was detected on the routine 45-day TEE. Unfortunately, no further surveillance imaging was conducted until the patient was symptomatic 10 months after implantation. The lack of follow-up monitoring may have delayed thrombus discovery.

It would be prudent to consider additional follow-up imaging beyond the 3-month mark, especially for high-risk patients (e.g., those with prior thromboembolism, large atria, or interrupted anticoagulation therapy). The optimal duration and frequency of post-LAAO surveillance imaging remain unknown. TEE or cardiac CTA is a reasonable approach 45 days to 3 months after implantation. Therefore, clinicians and patients must remain vigilant for late thrombi in the years following LAAO with a tailored strategy for each patient’s risk profile. Further research is needed to refine the post-occlusion surveillance protocols and anticoagulation strategies to prevent late thrombotic complications.

When DRT is identified, the cornerstone of management is the initiation or reinstitution of anticoagulants. Historically, vitamin K antagonists, such as warfarin, have been the most widely used treatment for DRT. Clinicians aim for a higher therapeutic INR range (2.5–3.5) to ensure effective thrombus resolution.^[7,21]^ In our patient, warfarin therapy (target INR: 2.5–3.5) over approximately 8 weeks successfully resolved the thrombus. Currently, there is no definitive consensus on the optimal anticoagulation agent or the duration of DRT. The management is typically guided by observational data and patient-specific factors.

Emerging evidence suggests that non-vitamin K antagonist oral anticoagulants (NOACs) are effective alternatives to warfarin. A recent multicenter registry reported no significant difference in thrombus resolution rates between initial treatment with warfarin *vs* NOACs therapy. In cases treated with vitamin K antagonists, 80% of DRT were resolved, whereas 75% of DRT cases resolved after NOACs treatment.^[20]^ Similarly, another series documented overall DRT resolution in 79% to 80% of cases by 3 months regardless of treatment (including heparin, warfarin, or NOACs).^[22]^ These data indicate that NOACs (such as rivaroxaban or apixaban) can be considered for DRT management, especially in patients who cannot tolerate warfarin or who have difficulty maintaining a therapeutic INR.^[20]^

Guidelines generally recommend continuing anticoagulation for at least 3 months after DRT is detected with repeat imaging to confirm thrombus resolution before considering tapering therapy.^[1,4,23]^ Some clinicians will pursue a higher target INR (e.g., 2.5–3.5) for warfarin therapy in DRT cases to maximize the chance of thrombus resolution, although the patient’s bleeding risk must be considered. The benefits of thromboprophylaxis must be balanced against the risk of hemorrhage. The regimen must be tailored to individual patient risk factors and tolerances.^[23]^

Another consideration is the improvement in device technology, which may reduce thrombus formation. Watchman FLX, a newer-generation LAAO device, incorporates design modifications to minimize leaks and thrombogenic surfaces. Notably, FLX has a polymer cap that fully covers the metal hub of the previous model, providing a smooth, recessed atrial surface and eliminating the exposed connector pin as a nidus for thrombus attachment. The FLX device is more conformable and requires design modifications (e.g., a different row of anchors) to achieve a more complete seal of the appendage ostium. Early clinical experience has indicated that these design changes have yielded benefits. Comparative studies showed that the Watchman FLX device significantly lowered the rates of PDL at 12 months compared with the original Watchman 2.5 device.^[9]^ Furthermore, initial reports have suggested a lower incidence of DRT with FLX.^[24]^ In a dual-center study, Watchman FLX was associated with fewer DRT occurrences than the first-generation device, along with improved LAA sealing.^[25]^

While long-term and randomized data are still forthcoming, these findings align with the expectation that a fully endothelialized, well-seated occluder is less prone to thrombosis. Other newer occluder systems, such as Amplatzer Amulet, which has a disc-and-lobe configuration and no exposed metal on the atrial side, may have low DRT rates, although no significant difference was shown in a randomized, controlled trial.^[13,26]^

Careful device selection and implantation techniques, along with advancements in occluder design, are important steps to minimize residual leaks and thrombosis risk. Future innovations in LAAO technology should focus on enhancing device endothelialization and eliminating exposed surfaces to reduce thrombotic complications further.

This report has several limitations. First, as a single case report, it cannot establish causality or be generalized to broader patient populations. Second, the frequency and timing of imaging follow-up may have influenced the detection and characterization of thrombus formation and resolution, thereby limiting the full understanding of disease progression. Third, the adjustment of antithrombotic therapy (i.e., discontinuation of rivaroxaban and subsequent initiation of high-intensity warfarin) reflected individualized clinical decision-making and may not be applicable to all patients. Finally, although TEE and cardiac CTA provided critical diagnostic information, both modalities have inherent limitations in sensitivity and interpretation. Larger prospective studies are warranted to validate the clinical observations and therapeutic implications highlighted in this case.

## 4. Conclusion

This case highlights that minimal PDL can predispose patients to late thrombus formation after LAAO, particularly when the duration of post-implantation anticoagulation therapy is short. Prompt and intensive anticoagulation therapy successfully treated the DRT. In our patient, high-intensity warfarin therapy led to complete thrombus resolution, without any stroke or embolic complications. This outcome reinforced the importance of strict imaging surveillance after LAAO and diligent management of any detected thrombi. It also underscores the need for optimized device placement and appropriate device choice to achieve a complete LAA seal. Ongoing improvements in device design are promising for reducing the incidence of DRT. Ultimately, managing patients after LAAO requires a delicate balance between preventing stroke and preventing bleeding. Vigilant follow-up and timely intervention are critical to achieve favorable outcomes in the face of late complications.

## Author contributions

**Conceptualization:** Yung-Lung Chen.

**Data curation:** Yung-Lung Chen.

**Supervision:** Chiung-Jen Wu, Hui-Ting Wang.

**Writing – original draft:** Yi-Ting Chen.

**Writing – review & editing:** Chiung-Jen Wu, Hui-Ting Wang.
